# Leveraging Africa’s underutilized crops to combat climate change, water scarcity, and food insecurity in South Africa

**DOI:** 10.1038/s41598-025-03853-4

**Published:** 2025-06-03

**Authors:** Abiodun Olusola Omotayo, Abeeb Babatunde Omotoso, John Awungnjia Asong

**Affiliations:** 1 Food Security and Focused Area Research Group, Faculty of Natural and Agricultural Sciences, North-West University, Mafikeng, South Africa; 2 Unit for Environmental Sciences and Management, Faculty of Natural and Agricultural Sciences, North-West University, Potchefstroom, South Africa; 3 Faculty of Plant and Environmental Sciences, Oyo State College of Agriculture and Technology, Igboora, P.M.B. 10, Nigeria

**Keywords:** Agro-biodiversity, Agricultural innovation, Dietary diversity, Ethnobotany, Resource value, Environmental health, Health occupations

## Abstract

This study aims to integrate underutilized crops (UCs) into the food system to address climate change impacts, and food -water insecurity. UCs have immense potential to mitigate food shortages, yet their role remains largely unexplored in mainstream agricultural and food security strategies. A multidisciplinary approach using social psychology, resource-based theory (RBT), and a new ecological paradigm was used to investigate factors influencing UCs adoption and their potential contribution to water and food insecurity in South Africa. The water poverty index (WPI) and household food insecurity access score (HFIAS) were used to determine the water and food insecurity status of rural households. The study found that UCs cultivation was driven by awareness, access to extension advisory services, and climate information. The findings indicate that adopting UCs significantly improves water and food insecurity in South Africa. Consequently, households that integrated UCs into their farming systems experienced higher WPI scores, reflecting improved water availability and conservation, as UCs require less water than conventional crops. Likewise, lower HFIAS values suggest that UCs enhance food insecurity by diversifying diets, stabilizing food access, and reducing seasonal hunger. Statistically, households in Limpopo, Mpumalanga, and North-West provinces who adopted UCs saw 25.18 (21%), 31.03 (26%), and 28.77 (24%) rise in WPI and HFIAS compared to those who did not embrace UCs, respectively. These results highlight the potential of UCs as climate-resilient crops that mitigate water scarcity and food insecurity, making them a viable strategy for enhancing rural livelihoods amid climate change. Therefore, prioritizing UCs cultivation could build more resilient agricultural systems, address water scarcity and improve food security.

**Supplementary Information:**

The online version contains supplementary material available at 10.1038/s41598-025-03853-4.

## Introduction

Global food security is progressively dependent on fewer than 30 plant species, with a great proportion of the population’s food needs relying on three major crops, that is, rice, maize and wheat^1,2^. These major or commodity crops have also been heavily researched, and, in some countries, their production is subsidised by governments^3^. With climate change predicted to result in an increased frequency of climate extremes^4,5^, it becomes interesting when considering the possible impact this will have on agriculture and food security^3,6^. The fate of the major crops cannot be guaranteed, and it is expected that diversification of food systems with currently underutilised crops (UCs) will relieve climate change threat^7^.

Interestingly, UCs are seen as coping strategies for food insecurity under persistent climate change and variability conditions^8–10^. They are plant species that have not been classified as major food crops but possess significant economic and ecological potential^11^. These crops are often locally important for food security, nutrition, and resilience against climate change, yet they remain under-researched and underexploited compared to staple crops like maize, rice, and wheat^3,12,13^. They are mainly restricted to small-scale farming systems and have been documented to be resilient against extreme environmental conditions, including drought and heat stress^7,14,15^. However, limited research has been conducted to understand the potential of UCs, with many of them cultivated only locally, without improved varieties^12,16^.

Noteworthy, South Africa faces a multidimensional crisis of climate change, water scarcity, and food insecurity, which disproportionately affects rural communities and smallholder farmers^5^. Notably, rising temperatures, erratic rainfall, and prolonged droughts have led to declining agricultural productivity, reduced water availability, and increased household food insecurity^16^. Conventional staple crops such as maize and wheat struggle to cope with these climatic challenges, making the diversification of agricultural systems an urgent priority. Despite their climate resilience and high nutritional value, UCs remain neglected in South Africa’s mainstream agricultural policies and food systems. Many indigenous crops, such as sorghum, millet, amaranth, Bambara groundnut, cowpea, and African yam bean, are well-adapted to low-input farming systems, yet they continue to be underexploited due to limited research, weak value chains, and market biases favouring commercial crops^11,17^.

Consequently, there is a need for a paradigm change away from the current streamlined food system that is dominated by a few crops to a more diverse system that embraces agro-biodiversity. There is, therefore, a need to further consolidate these UCs with the drought-tolerant and nutrient-dense, and food security attributes in South Africa. Subsequently, this article conducts an appraisal of the current status of some selected UCs and identifies their potential contribution to addressing climate change water and food-nutrition security in South Africa. This was achieved through the modelling of key determinants by applying socioeconomic, ethnobotany, institutional, environmental and farm-level approaches and correcting for selectivity bias stemming from both observed and unobserved heterogeneity. A key focus of the article is to raise awareness of the potential of the UCs as a drought-tolerant and nutrient-dense crop for a food and water-secure South Africa.

## Resource-based theory (RBT) and underutilized crop (UCs) species

The Resource Based Theory (RBT) emphasizes the critical role of resources in a firm’s ability to sustainably gain an advantage above competition^18–20^. The theory has undergone evolution due to the contributions of numerous scholars but has been critiqued for its limitations and weaknesses. Some issues include inappropriate definitions, overgeneralization, and construct validity^21,22^. Additionally, studies identified conceptual deficiencies and logic problems in the"strategically valuable resources"and framework for identifying resources as sources of sustained competitive advantage^23–25^. The core proposition of the RBT, which describes resources as the source of sustained competitive advantage, is flawed as it precludes the use of the scientific method in Resource-based view (RBV) research^23,26^.

Edith Penrose’s work significantly influenced the RBT, stating that a firm’s resources influence its growth and that sustainable development can only be achieved by effectively and efficiently employing all available resources^27,28^. One such resource is the underutilized crop (UCs), which are essential parts of agrobiodiversity and have been overlooked by researchers, agricultural officers, policymakers, and producers. UCs are largely restricted to smallholder farming areas and have several benefits, including nutritional, medicinal, economic, and environmental benefits^7,12^. However, research on UCs in Africa and South Africa in particular has generally not received the necessary attention from stakeholders. While there is some evidence of the value of UCs to rural households in some parts of the country, more research effort is needed.

Intriguingly, the core principle of the RBT states that a strategic resource is critical to the attainment of competitive advantage or the success of an organization or state^29,30^. UCs possess unique agronomic properties that make them thrive in diverse ecological niches and under unfavourable environments like poor soils and drought^12^. They are also useful substitutes in case of crop unavailability, inimitable and non-substitutable^17^. UCs as plant resources must be given the urgent attention, they deserve to address food and water insecurity among indigenous or rural communities in South Africa. UCs are a valuable resource that should be given the attention it deserves to ensure its continued use and success in the agriculture sector.

### Conceptualizing the underutilized crops’ (UCs) resource advantage(s)

**Fig. 1:**
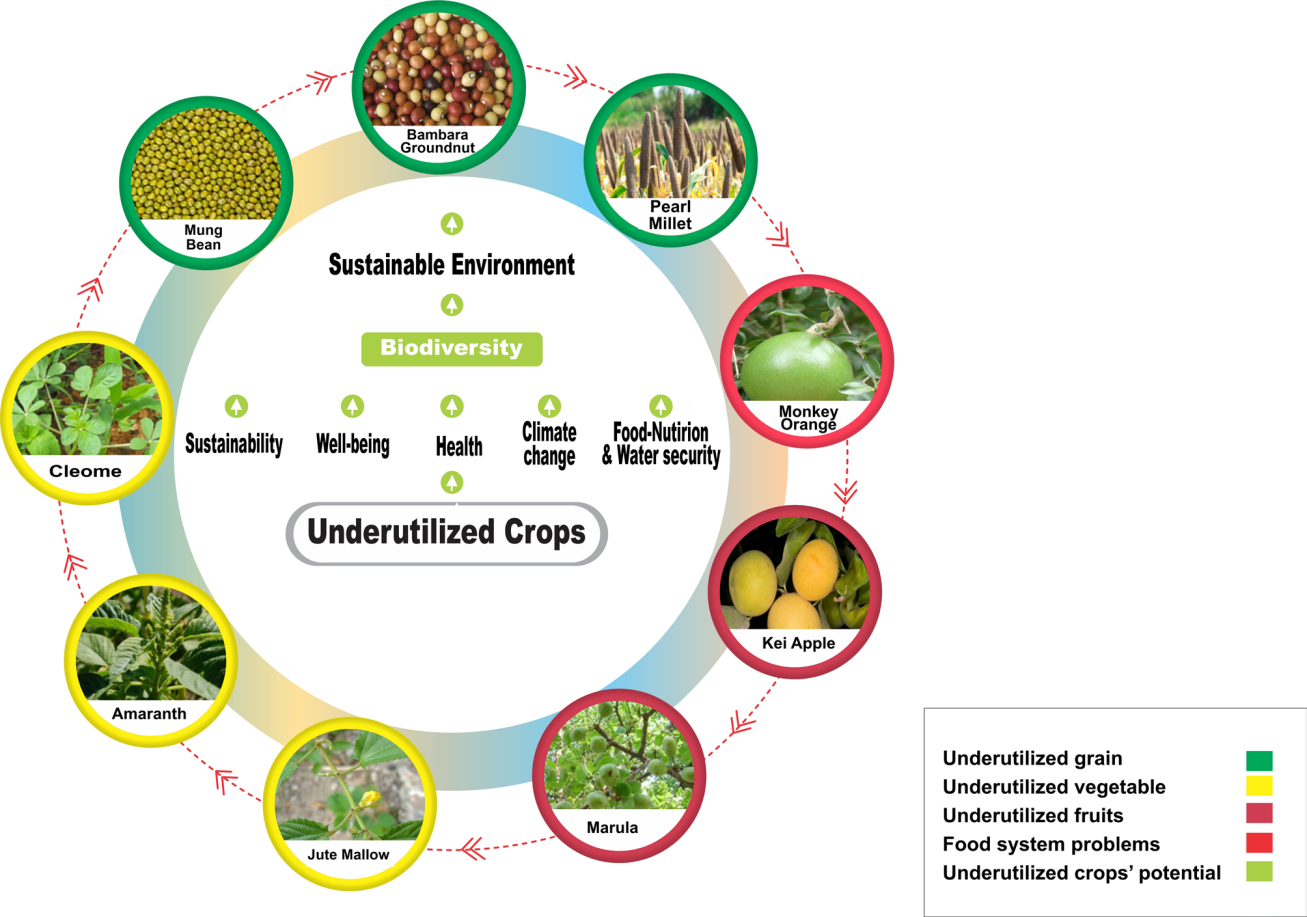
Schematic presentation of underutilized crops’ (UCs) potential to transform the food system.

Mahoney^31^, emphasizes the importance of effective resource utilization in a firm’s success, not just its presence of internal resources. UCs play a crucial role in enhancing local livelihood, nutrition, and food-water security among indigenous communities in developing economies^5,32^. However, their cultivation and use are constrained by factors such as climate change impact, lack of legal framework, market forces, pesticide abuse, land use change, and loss of traditional knowledge of UCs^3^. This research agrees with Mahoney’s perspective, stating that a political will is critical to guarantee economic value or results. Policy action is needed to ensure the effective cultivation, utilization and integration of UCs into the country’s food basket, as food nutrition and water insecurity (Fig. 1) are also a development issue.

In South Africa, food-nutrition and water insecurity remain a major challenge especially in the marginalised rural communities^16,32^. Research studies^33,34^, suggested that household food security is mainly a rural phenomenon, but interventions have not yielded the expected results. Meanwhile, UCs cultivation and integration are still confronted by problems such as climate change, water insecurity, diseases, insufficient land use, increased biofuel use, poor infrastructure and inadequate research funds towards UCs cultivation^7,35^. It is therefore imperative to adopt more innovative and strategic approaches to address this issue. Drawing from the RBT principles on resource use, two key resources should be identified: UCs resources and policy resources.

Effective and efficient exploitation of these resources can help the agricultural sector achieve a sustainable environment, biodiversity, livelihood and improved welfare, especially among rural households^9,13^. A deliberate strategy to integrate UCs into the national food basket is necessary, as there is currently no comprehensive policy detailing a strategy to facilitate the integration. We believe that a multidisciplinary approach that employs social psychological, resource-based theory (RBT), and a new ecological paradigm will aid the integration of UCs through necessary policy action constitutes the two key resources available to the agricultural sector in South Africa in addressing rural food, nutrition and water insecurity (Fig. 1).

## Research methods

### Study area and design

The research was done in three selected provinces out of the nine provinces of South Africa, namely the North West, Mpumalanga, and Limpopo provinces. The selection of the research location was determined by the social dynamics and low socio-economic level of the residents. Specifically, income level and population diversity, including ethnic and cultural heterogeneity, was a key consideration, as it influences social interactions and resource access^32^. These provinces serve as cultural and historical hubs in South Africa, deeply embedded in the traditions and heritage of indigenous communities^36^. Additionally, they share interconnected histories of resource-based livelihoods, indigenous knowledge systems, and food production, making them crucial for studying the role of UCs in addressing climate change, water, and food insecurity. The three provinces are situated at a latitude of 22° south of the Equator and a longitude of 28° east of the Greenwich meridian. Notably, these provinces encompass a total area of 307,131 km^2^, which accounts for about 25.17% of South Africa’s whole surface area^37^. The mean annual precipitation of about 470 mm, with the majority of rainfall occurring during the summer season from October to April^38^. The study location has a subtropical climate, with an average temperature ranging from 26.2 to 28 °C and a winter temperature of 26.2 °C^39^. Households in the study area engage in various climate change adaptation practices such as crop rotation, mulching, crop diversification, agroforestry and enterprise diversification, which involves the production of crops as well as the rearing of animals. The primary agricultural commodities cultivated in the selected provinces are maize, sunflower, and potato, as well as UCs such as mung bean, bambara groundnut, monkey orange, cleome and amaranth.

### Households survey data

This study is based on three cross-sectional surveys carried out in the North West, Mpumalanga, and Limpopo provinces with comparable aims of comprehending the cultivation of UCs by farmers and its impact on food and water insecurity in rural regions of South Africa. The surveys used a multistage sampling approach. Initially, a deliberate selection was made of nine representative districts (3 in each province) known for UCs cultivation. The second stage involves the selection of 25 villages randomly from these districts (known for UCs cultivation), including 8 villages in the North-West, and 9 and 8 villages in Mpumalanga and Limpopo, respectively. The villages were designated as the principal sample unit, from which rural households were randomly selected.

Lastly, a random selection of 13 households in these villages while a total of 320 households were successfully interviewed from the three provinces of South Africa. The interviews were conducted by a team of enumerators who underwent comprehensive training covering survey methodology, ethical research practices, and data collection techniques. Additionally, emphasis was placed on ethical considerations, including obtaining informed consent and ensuring respondent confidentiality. The training was conducted by principal investigators in collaboration with the district extension officer to ensure both theoretical and practical competence. Enumerators had at least a bachelor’s degree in fields such as agricultural economics, social sciences, or statistics, with preference given to those with prior fieldwork experience. Proficiency in local languages was an added advantage for effective communication. This rigorous training ensured that data collection was accurate, ethical, and culturally appropriate. The survey gathered data on a range of farm and household attributes, including the age and educational attainment of the household head, household size, off-farm employment, and access to institutional services such as agricultural extension, credit facilities, and membership in agricultural cooperatives, UCs identification and documentations, climate change and food-water security.

### Empirical estimation

#### Adoption impacts of underutilized crops on water and food insecurity – Multinomial endogenous switching regression (MESR) model

Following^40,41^, the MESR was chosen as an impact model because it can mitigate any bias that might obscure the results. This model is partitioned into two sectors. The first segment of the model addresses the issue of endogeneity, which arises from self-selection using a multinomial logit regression. This determines the farmers’adoption decisions in respect to UCs. For this study, farmer’s decision to cultivate at least one UCs was considered to be a dummy variable (assuming the value of 1 as an adopter and 0 as a non-adopter). The second section modelled the determinants of water and food insecurity of adopters of UCs and presented in Table 7 and Table 8. Farmers would be more likely to adopt UCs if the expected benefit of adopting UCs outweighs their non-adoption of it. Let U_j_, and U_i_ denote that the expected benefits of adoption and non-adoption, respectively (latent variables U_j_ – U_i_ > 0) are greater than non-adoption^42,43^. The explicit form of MESR assumed the following values:

1 $${\text{Y}}_{{\text{j}}} \, = \,0 \, \left( {{\text{non}} - {\text{adopters}}} \right):{\text{ Y}}_{{\text{j}}} {\text{K}}\, = \,{\text{X}}\prime \beta_{{\text{j}}} {\text{K}}\, + \,{\text{U}}_{{\text{j}}} {\text{K if D}}_{{\text{j}}} \, = \,0$$

2 $${\text{Y}}_{{\text{j}}} \, = \,{1 }\left( {{\text{adopters}}} \right):{\text{ Y}}_{{\text{j}}} {\text{A}}\, = \,{\text{X}}\prime \beta {\text{ A}}\, + \,{\text{U}}_{{\text{i}}} {\text{A if D}}_{{\text{j}}} \, = \,{1}$$

Where Y_j_A and Y_j_K are the dependent variables for an adopter and a non-adopter of UCs, respectively, while X′ is the collective symbol for the vectors of the explanatory variables.

#### Robustness check

Following^17,40^, we used a selection instrument to acquire a more robust identification. Consequently, we used access to extension training on improved agricultural production techniques and credit as our selection criterion. Notably^17,40^, have found that access to extension training for improved agricultural production practices typically involves practical training sessions and demonstrations. These activities allow farmers to acquire the necessary skills to effectively implement and utilize agricultural innovations. The validity of the proposed tool (access to extension training on improved agricultural production methods and credit), was confirmed by a falsification test. The results from Table 6 revealed that the selected instrument has a crucial role in affecting the decision-making of farmers who adopt UCs.

#### Measurement of outcome variables

We have 3 output variables: climate change adaptation strategies (such as crop rotation, mulching, crop diversification, drought-tolerant varieties, and agroforestry), water poverty index (water security) and household food insecurity access score (food insecurity). This study utilized the water poverty index (WPI) to measure water security. Following^44,45^, the WPI denotes the time and effort required to access water for household needs which involve the aggregate of water availability, access to safe water, and sanitation. Thus, if WPI = 100, the household is water-secure while the household is water-insecure if WPI = 0. Household Food Insecurity Access Score (HFIAS) was used to measure the food insecurity status of the households. HFIAS were assigned based on responses to a standardized set of nine questions that assess the frequency and severity of food insecurity experiences within a household over a recall period of four weeks. Each question captures a progressively severe aspect of food insecurity, ranging from worry about food availability to going a whole day without eating. Following^16,33^, HFIAS was calculated as the sum of the response values across all nine questions, ranging from 0 (food secure) to 27 (severely food insecure).

### Analytical and estimation techniques

#### Endogenous switching regression (ESR) model

Endogenous self-selection of UCs farmers into adoption and non-adoption groups may result in biases from both observable and unobservable factors^40,46^. Following^16,47,^ the selectivity bias was modified using the ESR Model by implementing a logit model. Furthermore, ESR is one of the most effective methods for accounting for the influence of unobservable factors on both the adoptions and outcome variables^36^. The ESR framework is divided into two phases. To model the determinant of UCs adoption and elucidate the unobserved variability in the initial stage of the ESR model, a logit model was implemented. The following is the selection equation for the adoption decision:

3 $${\text{A}}_{{\text{i}}} \, = \,{\text{X}}_{{\text{i}}} \beta \, + \,\mu_{{\text{i}}} \quad {\text{given that Y}}_{{\text{i}}} = \left\{ {\begin{array}{*{20}c} {1 \,if\, {\text{Ai}}> 0} \\ {0\, otherwise} \\ \end{array} } \right.$$

Where Ai represents a binary variable indicating the adoption of UCs; β is a parameter vector to be estimated; X is a vector of rural farmers’socioeconomic and institutional characteristics that influence farmers’adoption decisions, such as access to extension training on improved agricultural production practices, credit, and climate information; and μ is the random error term. The ESR model considers selectivity bias and calculates the impact of UCs on outcome variables by analyzing the interaction between the adoption choice of UCs and explanatory variables^40,48^. The second stage involves combining an Ordinary Least Squares regression with a selectivity correction term derived from the first stage. This is done to analyze the relationship between outcome variables (WPI, and HFIAS) and a set of explanatory factors, taking into account the adoption decision of UCs farmers.

### Ethnobotanical information and analysis of the underutilised crops (UCs)

Frequency of Citation (FC): Based on the study of^49^, the frequency of citation (FC) of the plant species was calculated as follows:

4 $$FC\, = \,Np/N\, \times \,{1}00$$

where ^Np^ = number of times a particular species was mentioned; N = total number of times that all species were mentioned × 100.

### Ethical considerations

The authors confirm that the household survey was carried out in accordance with relevant guidelines and regulations. The research obtained ethical permission and approval from the ethics committee of the Faculty of Natural and Agricultural Sciences (FNAS), North West University, South Africa. All methods were performed in accordance with the relevant guidelines and regulations of the ethics committee. The ethic clearance was granted with a low-risk classification, as shown by ethical clearance certificate no: NWU-01243–19-S9. Prior to administering questionnaires in the research, permission to access the area was obtained from the North West Provincial Department of Rural, Environment and Agricultural Development (READ), South Africa. The ethnobotanical survey was undertaken with the explicit permission of all participants. We presented comprehensive information regarding the research, as well as the anticipated outcomes from both the researchers and the participants. Informed consent was obtained from all subjects and/or their legal guardian(s). These principles encompassed the concept of voluntary engagement and the ability for participants to exit at any moment. Throughout this investigation, the researchers and volunteers diligently upheld the principles of privacy, autonomy, dignity, and respect (Ubuntu).

## Results and discussion

### Farm households’demographic distributions and climate change adaptation strategies adopted

**Fig. 2:**
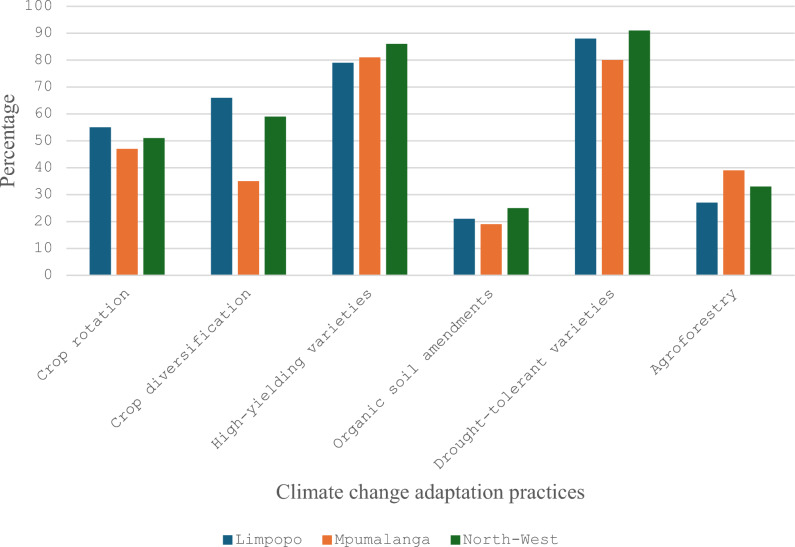
Climate change adaptation strategies adopted in the selected provinces of South Africa.

The various forms of climate change adaptation strategies (CCAS) in response to climate change by UCs farmers were crop rotation and diversification, high-yielding varieties, organic soil amendments, agroforestry and drought-tolerant varieties (Fig. 2). Studies by^17,40^ highlight that farmers growing indigenous and underutilized crops integrate crop rotation and diversification as key adaptation strategies to reduce vulnerability to climate extremes. Conversely, Table 1 presents the summary statistics of the variable employed in the MESR. The results showed that the average HFIAS, food group and water poverty index of the sampled households in South Africa (Table 1), were estimated at 4.45, 7.84 and 25.92, respectively.Additionally, the WPI of 33.52, 21.03 and 28.77 indicates a considerable water stress in Limpopo, Mpumalanga and North West provinces, respectively. The results shows a significant water insecurity in South Africa, mostly due to limited water supply and the time taken to source it. Existing studies by^32,50,^ confirmed that Limpopo and North-West provinces face severe water shortages, with rural households often relying on rainwater harvesting and distant communal water points.

**Table 1:** Descriptions and summary statistics of the variables used in the analysis.

Variables	Descriptions	Mean	Std. dev	Min	Max
**Outcome variables**					
HFIAS	An absolute food-secure household (HH) receives a score of 0, while a completely food-insecure HH receives a score of 27	4.45	2.72	1.92	18.31
Food groups	Measured by intake of food groups consumed by the HH	7.82	4.05	2.01	11.86
Water poverty index	Generated by combining factors that accurately represent the composite assessed	25.92	18.29	16.61	32.76
Treatment variable					
UCs cultivations	UCs adopted (Dummy; 1 if yes (cultivated UCs in last agricultural season), 0 if otherwise	0.617	0.336	0	1
**Socioeconomic characteristics**					
Age	Actual age of household head (HH)	46.12	24.21	19	72
Sex	Dummy; 1 = male, 0 = female	0.782	0.441	0	1
Household size	Total member of household	5.14	3.12	1	7
Marital status of HH	Dummy; 1 if married, 0 if otherwise	0.801	0,462	0	1
Educational status of the household head	Dummy; 1 if educated, otherwise	0.692	0.402	0	1
Years of experience	Actual year (continuous)	9.67	3.07	3	14
Land ownership	Dummy; 1 if yes (HH the ownership of the farmland), 0 if otherwise	0.567	0.345	0	1
Source of financing farm operation	Dummy; 1 if personally financed by HH 0 if otherwise	0.886	0.502	0	1
**Interested variables**					
Off-farm income	1 if yes (HH engaged in off-farm income generating activities), 0 if otherwise	0.674	0.361	0	1
Total farm size for other crops cultivation	Actual farm size used for other crop (such as maize, potatoes and vegetables) cultivation (Continuous)	6.08	2.91	1	12
Total land used for UCs cultivation	Actual land area used for UCs cultivation (Continuous)	3.31	1.09	2	6
Enterprise diversification	Dummy; 1 if yes (HH engaged in livestock farming), 0 if otherwise	0.782	0.220	0	1
Membership of cooperative society	Dummy; 1 if yes (HH member of coop society), 0 if otherwise	0.591	0.186	0	1
Access to credit	Dummy; 1 if yes, 0 if otherwise	0.483	0.192	0	1
**Institutional variables**					
Information on climate change	Dummy; 1 if yes, 0 if otherwise	0.782	0.220	0	1
Contact with extension agents	Dummy; 1 if yes, 0 if otherwise	0.608	0.298	1	7
Participate in extension training	Dummy; 1 = Yes to HH participate in extension training on improved agricultural production practices, 0 = otherwise	0.673	0.411	0	1
Perceived benefit of UCs	Dummy; 1 if yes, 0 if otherwise	0.775	0.306	0	1
**Information sources on UCs benefit and cultivation**					
Community meeting	Dummy; 1 if yes, 0 if otherwise	0.702	0.417	0	1
Flyers/Posters	Dummy; 1 = Yes, 0 = otherwise	0.364	0.115	0	1
Radio/Local newspaper	Dummy; 1 if yes, 0 if otherwise	0.474	0.190	0	1
Smartphone/Internet	Dummy; 1 if yes, 0 if otherwise	0.665	0.417	0	1

Interestingly, the dataset extensively analyzed several household characteristics that were linked to the adoption decisions of UCs by rural farmers. The attributes of rural farmers were taken into account when modifying socioeconomic factors (such as gender, age, family size, farm experience, land ownership, and farm size), and institutional factors (climate information, information on the benefit of UCs, and participation in extension training). Table 2 revealed that the average age of the household head in South Africa was 46 years. This implies that household heads would have extensive knowledge of traditional farming techniques and the benefits of UCs. This experience would influence their openness and readiness to adopt and use UCs^16^. The majority of family heads have achieved literacy, as seen in Table 2. Furthermore, farmers who possess advanced human capital, as shown by their extensive education, have the knowledge and skills required to make educated judgements on improved agricultural production practices and adoption decisions which would significantly influence their water and food insecurity status^16^. Moreover, a significant number of rural farmers participated in extension training sessions that specifically emphasized improved agricultural methods. Additionally, extension training plays a vital role in spreading information, offering technical assistance, and enabling farmers to make educated decisions about agricultural innovation^46^.

**Table 2:** Summary statistics of UCs adopters in selected provinces of South Africa.

Variables	Limpopo		Mpumalanga		North West	
	**Mean**	**Sth. dev**	**Mean**	**Sth. dev**	**Mean**	**Sth. dev**
**Outcome variables**						
HFIAS	3.15	1.55	5.06	2.11	4.78	1.89
Food groups	7.89	3.05	6.71	3.22	8.06	4.49
Water poverty index	33.52	17.90	21.03	11.04	28.77	9.82
**Treatment variable**						
UCs cultivations	0.805	0.492	0.871	0.436	0.934	0.398
**Socioeconomic characteristics**						
Age	45.34	21.17	47.05	20.19	48.93	22.09
Household size	5.12	3.95	6.11	3.03	5.22	3.46
Marital status of the household head	0.783	0.351	0.674	0.285	0.889	0.407
Educational status of the household head	0.801	0.482	0.673	0.275	0.776	0.296
Years of experience	11.89	4.80	9.52	12.53	9.71	3.04
Land ownership	0.611	0.262	0.385	0.227	0.725	0.341
Source of financing farm operation	0.683	0.221	0.459	0.369	0.974	0.512
**Interested variables**						
Off-farm income	0.782	0.240	0.463	0.114	0.863	0.271
Total farm size for other crops cultivation	8.17	2.98	7.63	3.09	5.67	3.41
Total land used for UCs cultivation	2.72	1.45	4.01	1.91	3.37	1.81
Enterprise diversification	0.821	0.228	0.519	0.251	0.983	0.571
Membership in a cooperative society	0.651	0.289	0.713	0.382	0.882	0.401
Access to credit	0.542	0.351	0.674	0.273	0.582	0.250
**Institutional variables**						
Information on climate change	0.617	0.350	0.723	0.362	0.583	0.201
Contact with extension agents	0.731	0.385	0.719	0.361	0.812	0.594
Participate in extension training	0.388	0.073	0.509	0.205	0.718	0.307
Perceived benefit of UCs	0.401	0.318	0.618	0.227	0.274	0.141
**Information sources on UCs benefit and cultivation**						
Local newspaper/community meeting	0.398	0.203	0.586	0.228	0.273	0.115
Flyers/Posters	0.739	0.374	0.680	0.319	0.472	0.207
Radio	0.774	0.492	0.927	0.504	0.305	0.135
Smartphone	0.583	0.221	0.982	0.539	0.289	0.117

Intriguingly^41^, posited that investments and savings are of utmost importance in the first phases of technology adoption. These funds may be used to increase capital and decrease susceptibility to the negative consequences of climate change. As a result, they would have an influence on the variety of food consumed and security, and the scarcity of water for rural households^17^. In the same vein, the model included agricultural enterprise diversification which includes the administration of animal, poultry, and other small-scale livestock farms to ascertain its potential impact on the adoption of UCs in South Africa. Subsequently, diversifying agricultural activities mitigates risk and enhances the probability of consistent income for households, hence facilitating the adoption of capital-intensive technologies and reducing the volatility of agricultural operations^5^. This fosters regular food intake within families and reduces vulnerability to the negative impacts of climate change^16^. Consequently, a satisfying relationship is anticipated.

### Underutilized crops (UCs) diversity

**Fig. 3:**
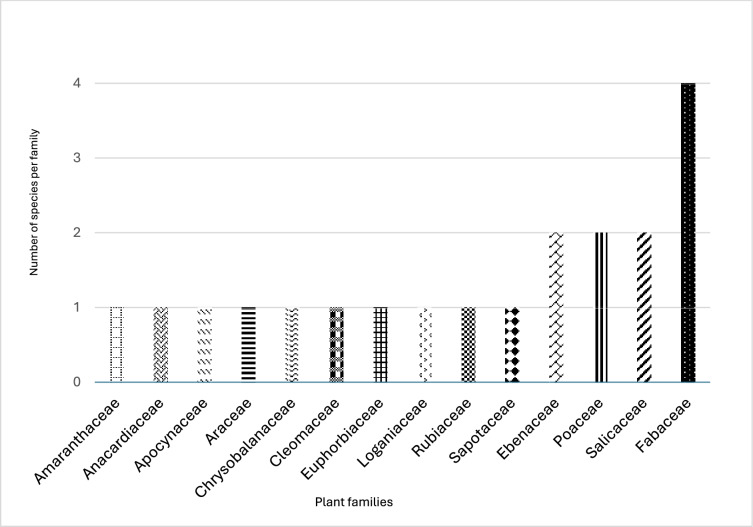
Families of UCs identified in three selected provinces of South Africa.

The flora from the selected provinces is rich and provides diverse useful species. The study documented 21 underutilized crops (UCs) distributed among 14 families (Fig. 3 and Table 3). Fabaceae and Salicaceae families had the highest proportion of edible species in the three provinces. The result confirms with^13,22^ who posited that the Fabaceae family has been mentioned as one of the plant families consisting of several plant species used in food amongst different indigenous communities in Southern Africa^51,52^. Furthermore, existing literature supports the popularity of Fabaceae for their therapeutic efficacy and nutritional composition^53,54^. Additionally, Fabaceae family members are globally significant culturally and economically, providing traditional medicines, food, timber, garden ornamentals, dyes, fibres, fuels, gums, and insecticides^50,52^. Based on their FC values, which ranged from approximately 0.3–90%, the most popular UCs used by the participants were *Carissa macrocarpa* (Eckl.) A.DC (92.8%), *Manihot esculenta crantz* (92.5%), *Pennisetum glaucum* (L.) (92.1%), *Amaranthus spp* (92.1%), and *Mimusops zeyheri sond* (92.1%) (Table 3).

**Table 3:** Ethnobotanical information on the UCs consumed in the selected provinces of South Africa. Frequency of citation (FC).

Scientific name & Family name	^$^Vernacular name	Plant part	Usage	Nutritional compositions	Reference	*O	N	FC
**Grains**								
*Sorghum bicolor* (L.) Moench Poaceae	Mabele (T), Amazimba, Amabele;	G	Staple food and sorghum beer	S. bicolor is mainly made up of carbohydrates, followed by proteins, lipids, crude fibre, and ash; it also provides minerals such as phosphorus, potassium, magnesium, calcium, iron and zinc; vitamins A, D, E and B complex	^55^	D	277	86.5
*Pennisetum glaucum* (L.) R.Br Poaceae	Nyalothi, Ntweka, Amabele, Unyaluthi, Unyawoti,	G	Staple food	Carbohydrates, amylose, sucrose, glucose, protein, total dietary fibre, lipids, and fatty acids [total lipid, palmitic, linoleic, oleic, and stearic acid]	^56^	D	295	92.1
*Vigna unguiculata* (L.) Walp Fabaceae	Cowpea (E); Dinawa, Dinaba, Munawa, Imbumba, akkerbone	L & G	Staple foods and essential sources of dietary protein	Whole grain, leaves, aerial parts, and decorticated grain are rich in carbohydrates, proteins, lipids, minerals, total dietary fibers, and vitamins	^57^	D	200	62.5
*Vigna radiata* (L.) R.Wilczek Fabaceae	Dithlodi (T), Mung bean (Eng); Mgcenga (siSwa);	L and G	Staple foods are essential sources of dietary protein and carbohydrate	V. radiata is a plant rich in carbohydrates, proteins, copper, iron, phosphorus, potassium, selenium, manganese, zinc, and calcium	^58^	D	281	87.8
*Vigna subterranea* (L.) Verdc Fabaceae	Bambara groundnuts	L & G	Staple food and snack	Crude protein, fats, iron, sodium, potassium and zinc	^59^	D	257	80.3
**Vegetables**								
*Amaranthus sp* Amaranthaceae	Thepe (Ts); Amaranthus (E);Infino (Z)	L	Staple food and medicinal use	It is rich in flavonols, less rich in tannins, protein, ash content, crude protein, crude lipid, crude fibre, and carbohydrate, sodium, potassium, calcium, magnesium, iron, zinc, phosphorus, and vitamin	^60–67^	W	295	92.1
*Cajanus cajan* (L.) Millsp. Fabaceae	Pigeon bean (E); Dinawa (Ts)	G & L	Staple food and vegetable	High protein, amino acids	^68^	D	26	8.1
*Corchorus olitorius* L Malvaceae	Wild jute, wildejute, Thelele, Delele, Gushe	L	Used as vegetable	Rich in minerals (Calcium, Iron) and vitamins B1, B2, Folic acid and E	^69^	D	190	59.3
*Cleome gynandra* L Cleomaceae	Lerotho (Ts); Cat’s whiskers, Cleome, African cabbage (E); Oorpeultjie (A)	L	Used as vegetable	Vitamins (A and C) and minerals (Calcium and Iron)	^70,71^	D	190	59.3
*Colocasia esculenta* (L.) Schott Araceae	Amadumbe, Amadombie, Amadombi, Mufhongwe (Z)	R	Used as vegetable	Tubers supply easily digestible starch and are known to contain substantial amounts of Protein, vitamin C, thiamine, riboflavin and niacin	^72–74^	D	183	57.1
*Manihot esculenta* Crantz Euphorbiaceae	Muthupula (Ts); Ntsumbula (Tso); Umdumbula Othobola (Z)	R	Used as vegetable	Cassava roots provide energy, while leaves offer protein, vitamins, and minerals. Roots contain crude fat, proteins, and carbohydrates like sucrose, glucose, fructose, and maltose	^75,76^	D	293	91.5
**Fruits**								
*Carissa macrocarpa* (Eckl.) A.DC Apocynaceae	Natal plum, big num-num (Eng.); grootnoem-noem (A); Amatungulu (Z)	F	Freshly eaten, prepared fruit salad and jam	The fruits of C. macrocarpa are high in important fatty acids, vitamin C, and several chemical elements including calcium, potassium, magnesium, iron, and copper	^7,11,75^	D	297	92.8
*Diospyros lycioides* Desf Ebenaceae	Monkey plum (E); bloubos (A); Lethanyu (T); Monkga-nku (S-Sotho);	F	Ripe fruits are edible	The leaves and the fruit contain protein, carbohydrates, fibre, unsaturated fat-rich lipids, minerals, vitamins, phosphorus, magnesium, calcium, iron, potassium, sodium, zinc, copper, and manganese	(Bagla et al.,2016; Mujuru et al., 2012)	W	178	55.9
*Diospyros simii* (Kuntze) De Winter Ebenaceae	Climbing Star-apple (E); Kraaibessie (Afr)	F	Edible fruit	NA	NA	D	2	0.62
*Dovyalis caffra* (Hook.f. & Harv.) Sim Salicaceae	Kei-apple (E); Kei-appel (A); Motlhono (Ns); Umqokolo (Z);	F	Fruits eaten raw and juice	High Vitamin C content	^78^	D & W	183	57.1
*Dovyalis zeyheri* (Sond.) Warb. Salicaceae	Wild apricot (E); Wilde-appelkoos (A); umNyazuma (Z); umQokokolo (X);Morethema (Ns)	F	Fruits eaten raw	Carbohydrate, crude fibre, protein, ash, moisture and fat	^79–84^	W	1	0.31
*Mimusops zeyheri* Sond Sapotaceae	Transvaal red milkwood (E); Moepel (A); Mmupudu (NS); umpushane (Z); Nhlantswa (T); Mubululu (V)	F	Fruits eaten raw and as beverages	Minerals and vitamins (A and C). Mineral elements potassium and calcium	^7,85,86^	W	295	92.1
*Parinari curatellifolia* Planch. ex Benth Chrysobalanaceae	Bosappel (A); Mmola (N. Sotho); Mbulwa (Tso); Mobola (Ts); Muvhula (V)	F	Fruits eaten raw	Protein, vitamin C, fat and ash contents, and minerals (calcium, iron, magnesium, and zinc)	^87^	W	178	55.9
*Sclerocarya birrea* (A.Rich.) Hochst Anacardiaceae	Marula (E); Morula (N-S); Mufula (V); ukanyi (Tso)	F	Fruits eaten raw and used as tea and beverages	The fruit contains considerable amounts of dietary fiber, protein, vitamins (A, B3, C, E and carotene), minerals, amino acids, and fatty acids	^88,89^	W	189	59.0
*Strychnos spinosa* Lam Loganiaceae	Corky-bark Monkey-orange (E); Kurkbasklapper (A); Morapa (Ns);	F	Fruits eaten raw	This plant includes dry matter, ash, crude protein, fat, fibre, Vitamin C, phosphorus, magnesium, calcium, iron, potassium, sodium, zinc, copper, and manganese	^90,91^	W	189	59.0
*Vangueria infausta* Burch Rubiaceae	Chirinda wild-medlar, (Eng.); Bosmispel, Blinkblaarmispel (A); Mobilo (Ts)	F	Fruits eaten raw	Vitamins and minerals	^92–97^	W	200	62.5

^$^Vernacular names: A = Afrikaans, E = English, Z = Zulu, Ns = Northern Sotho, Ts = Setswana, Tso = Xitsonga, X = Xhosa *Occurrence: W = Wild and D = Domestic. Plant parts; G = Grains, N = Number; NA = Not available Names for the plants were verified using the World flora online (http://www.worldfloraonline.org/).

### Use-categories of underutilized crops (UCs)

**Fig. 4:**
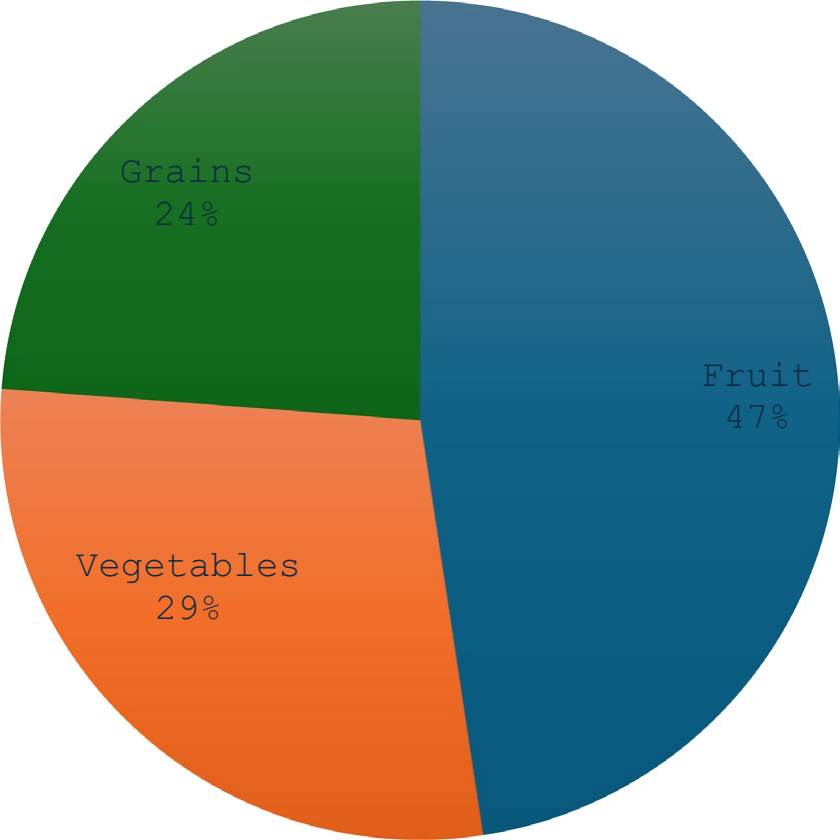
Use-categories for UCs in the three selected provinces of South Africa (n = 320).

This study classified underutilized crops (UCs) into three main categories: grains (5 species), vegetables (6 species), and fruits (10 species). The dominant life forms of UCs include shrubs, herbs, and trees, with 47% of fruit-bearing UCs growing in the wild, while 24% of grains are cultivated in ploughing fields and home gardens for vegetable production (Fig. 4). Grains are primarily cultivated in home gardens, where they are either ground into flour or consumed raw. Unlike conventional staple grains such as maize and wheat, these underutilized grains provide essential nutrients and are often more resilient to drought, pests, and poor soil conditions, making them ideal for climate adaptation strategies^11,17^. Studies^11,79^ confirm that a significant proportion of UCs are wild species, although some are domesticated in home gardens. Furthermore, the widespread edibility of these crops across diverse communities highlights the existence of shared indigenous knowledge on their use among different subsistence groups, cultures, and geographic regions^79,80,98^.

Consequently, the cultural and geographical diversity of UCs further underscores their significance in traditional food systems^13^. Their widespread edibility across various ethnic and communities suggests a rich body of indigenous knowledge regarding their identification, processing, and consumption^41^. This common knowledge base, shared across subsistence farming groups in different ecological zones, highlights the historical and socio-economic importance of UCs in ensuring food security, dietary diversity, and ecological sustainability. Moreover, increasing scientific validation of the nutritional benefits of UCs has the potential to influence policy decisions, promoting their integration into formal agricultural systems and national food security strategies^99^.

### Underutilized crops (UCs) parts

Participants in the study area utilized various UCs parts for food preparation, including leaves, roots, seeds, grains, and fruits. Among these, fruits were the most commonly harvested plant part (45%), followed by grains (27%), leaves (23%), and rhizomes (5%). The majority of these plants were consumed fresh, while some were used in both fresh and dried forms, and a few were exclusively prepared from dried materials. Similar trends have been observed in other regions of Southern Africa, where wild edible fruits are predominantly consumed raw in fresh form^13^. Although, wild edible plants continue to be an essential food source in South Africa, their utilization and management are increasingly threatened by anthropogenic factors, leading to a gradual erosion of indigenous knowledge^9^. This study highlights that while indigenous fruits remain the most frequently consumed edible plants, grains also play a critical role in household food-nutrition due to their cultural and dietary significance.

Additionally, leafy vegetables are gaining recognition for their substantial health benefits, including their ability to prevent serious illnesses and improve the nutritional status of vulnerable populations^22^. These vegetables are among the most affordable and accessible sources of essential nutrients, providing vitamins, minerals, amino acids, and other bioactive compounds necessary for overall health and well-being^100^.

### Adoption decision of underutilized crops and impacts on water and food insecurity

The estimates for the parameters that influence the adoption of CSA across the designated provinces of South Africa are presented in Table 4. The results are assessed against the baseline of non-adopters of UCs. Based on the Wald test results [χ2 (101) = 156.11, p = 0.001), the model exhibits a satisfactory fit to the data. Additionally, the results indicate that the coefficients are substantially different across the study locations. Furthermore, the second stage MESR on the determinant of the outcome (water and food insecurity) variables were presented in Table 7 and Table 8. The results presented in Table 4 indicated that the areas of land used for food crop cultivation in South Africa are directly correlated with the adoption of the UCs. This suggests that the size of land allocated for food crop cultivation in South Africa has a direct influence on the likelihood of adopting UCs. The correlation implies that as the cultivated land area increases, farmers are more inclined to incorporate UCs into their farming systems. This could be due to several factors, such as increased land availability allowing for diversification, reduced risk associated with experimentation, or the ability to allocate portions of land to alternative crops without jeopardizing staple food production. The results are consistent with^39,46^ which suggest that the probability of transitioning to enhanced technology in South Africa increases in direct proportion to the expansion of the cultivated agricultural area.

**Table 4:** First stage of MESR: Determinants of underutilized crops (UCs) adoption.

Variables	**Limpopo**		**Mpumalanga**		**North West**	
	**Coefficient**	**Robust SE**	**Coefficient**	**Robust SE**	**Coefficient**	**Robust SE**
**Socioeconomic variables**						
Age	0.2271***	0.0511	0.0053**	0.0021	0.1765	0.0643
Household size	0.0953***	0.0301	−0.0861	0.1231	0.0895***	0.0241
Marital status of the household head	−0.1085	0.0983	0.0951**	0.0534	−0.0251**	0.0101
Educational status of household (HH)	0.1984***	0.0320	0.0164**	0.0089	0.0191**	0.0052
Years of experience	0.0256***	0.0031	0.0017***	0.0002	0.0362	0.1632
Land ownership	0.0012***	0.0002	0.0334	0.0577	0.0033**	0.0017
Source of financing farm operation	0.0834	0.3921	0.0657**	0.0311	−0.3351	0.4511
**Interested variables**						
Off-farm income	0.0027**	0.0015	0.0044**	0.0022	0.2096***	0.0342
Total farm size for other crops cultivation	0.1063**	0.0662	0.0112***	0.0025	0.0185***	0.0041
Total land used for UCs cultivation	0.1165**	0.0562	0.0849*	0.0442	0.0763*	0.0337
Enterprise diversification	0.0341**	0.0126	0.2334*	0.1199	0.0085***	0.0021
Membership of cooperative society	0.0145**	0.0071	0.0051	0.0104	0.0050*	0.0028
Access to credit	0.0895***	0.0085	0.1175**	0.0520	0.0096*	0.0051
**Institutional variables**						
Information on climate change	0.0540*	0.0321	0.0931**	0.0417	0.1003*	0.0512
Contact with extension agents	0.0237	0.0402	0.1063**	0.0511	0.0882***	0.0301
Access to extension training	0.0053**	0.0026	0.1784**	0.0532	0.0335***	0.0051
Perceived benefit of UCs	0.0476***	0.0103	0.0933***	0.0095	0.0031**	0.0016
**Information source**						
Local newspaper/community meeting	0.6902	0.7647	0.0056**	0.0021	0.0044	0.0121
Flyers/Posters	0.0703*	0.0362	−0.0003	0.0018	0.9845**	0.4153
Radio	0.0262***	0.0023	0.0044**	0.0021	0.0458***	0.0036
Smartphone	0.1008*	0.0675	0.0201*	0.0105	0.0763	0.1905
Constant	0.7200***	0.1832	0.0038***	0.0011	0.0543**	0.0213

Note: ***, ** and * denotes significant at 1%, 5% and 10% respectively.

The study demonstrated a direct correlation between the literacy levels of household heads and the adoption of UCs in the three provinces (Limpopo, North-West and Mpumalanga). The likelihood of adopting UCs is increased by the presence of educated household heads, irrespective of their gender and provincial residence. Education is essential for farm families to understand the advantages of new agricultural technologies^16^. It accomplishes this by increasing their awareness and enhancing their decision-making process regarding the adoption of these technologies^17,41^. The adoption of UCs in the Limpopo and North-West provinces was positively correlated with the membership of household heads in agricultural cooperatives. This discovery corroborates the outcome presented in Table 2, which indicates that households in Limpopo and North-West are better off than their counterparts in Mpumalanga province in terms of water poverty index and food insecurity. Cooperative membership facilitates the acquisition of inputs, financing, and market access for members^10,34^.

The results also showed that, in South Africa, smallholder farmers with restricted credit availability exhibit a lower propensity to adopt UCs. As a result, the adoption of innovative agricultural technology requires a financial investment. Previous research studies^101,102^ have demonstrated that rural farmers are more inclined to adopt enhanced agricultural technologies when they have access to financing. Conversely, the result revealed that the likelihood of smallholder farmers in adopting agricultural technology (UCs) is significantly influenced by the availability of climatic information in South Africa. This research consistently revealed that the adoption of UCs was positively influenced by access to climatic information in South Africa, regardless of the provincial disparity among smallholder farmers. Consequently, this phenomenon may be attributed to the fact that the adoption and promotion of UCs were facilitated by the availability of information on the perceived benefit of UCs through media such as smartphones/internet, community meetings and radio and extension agents, which transformed into a better water and food security status.

In addition, the adoption of UCs is significantly influenced by the participation of household heads in off-farm income-generating activities, which implies that on-farm and off-farm activities are mutually supportive. By participating in off-farm income-generating activities, smallholder farmers can surmount financial constraints that may impede their adoption of new agricultural technology^41^. This also validates the selection of off-farm income as our instrumental variable in the endogenous switching model. Furthermore, the probability of smallholder farmers in South Africa with large family sizes adopting UCs increased substantially. The presence of an abundant labour supply from large family households, particularly for labour-intensive agricultural technologies like the integrated farming system and crop diversification, could be considered a contributing factor^10,103^.

### Average treatment (ATT) and heterogeneity effects estimation

This study aims to evaluate the effects of adopting underutilized crops (UCs) on the outcomes associated with water and food security (WPI and HFIAS) among rural farming households in South Africa. The MESR enables the attainment of expected outcomes related to WPI and HFIAS, subject to the adoption of UCs in their farming enterprise. Table 5 illustrates the average treatment effect and heterogeneity of UCs adoption on household outcome (WPI and HFIAS) variables in South Africa. Consequently, the heterogeneity effects, which account for the potential heterogeneity in the sample rural households, suggest that in the counterfactual scenario, rural households that implemented UCs would have produced significantly more than those that did not. This result emphasizes substantial heterogeneity factors that differentiate adopters as"better water and food secure households"from non-adaptors in the context of climate change.

**Table 5:** Impact (actual and counterfactual) of adoption of UCs on the outcome variables – HFIAS and WPI.

Outcomes		Limpopo			Mpumalanga			North West		
		**Adopt**	**Not adopt**	**Treatment effect**	**Adopt**	**Not adopt**	**Treatment effect**	**Adopt**	**Not adopt**	**Treatment effect**
**HFIAS**	Adopters	4.45*** (1.96)	6.41*** (2.43)	−1.96*** (0.073)	5.05** (2.11)	7.26** (3.91)	−2.21*** (1.07)	4.78*** (1.89)	6.61** (3.42)	−1.83** (0.95)
	Non-adopters	7.71* (4.68)	9.27*** (3.87)	−1.56*** (1.03)	6.27** (3.17)	8.99* (1.83)	−2.65** (1.37)	5.96*** (2.03)	7.33*** (3.67)	−1.37*** (0.42)
	Heterogeneity effects	−3.26*** (1.09)	−2.86*** (0.75)	0.40*** (0.14)	−1.22*** (0.43)	−1.73* (0.93)	0.44** (0.21)	1.18*** (0.53)	0.72*** (0.13)	0.46** (0.25)
**WPI**	Adopters	25.18*** (12.01)	23.25** (11.74)	1.93* (1.02)	31.03*** (11.04)	21.90*** (8.31)	9.13*** (1.17)	28.77 (19.82)	21.67*** (12.92)	7.10*** (3.71)
	Non-adopters	22.89** (11.08)	22.03** (12.83)	0.86*** (0.07)	33.52*** (15.75)	25.82*** (13.02)	7.70*** (2.05)	23.04* (12.33)	19.02** (9.11)	4.02** (2.13)
	Heterogeneity effects	2.29*** (0.32)	1.22*** (0.21)	1.07** (0.58)	−2.49*** (1.03)	−3.92** (1.96)	1.43 (0.98)	5.73*** (2.11)	2.65** (1.41)	3.08*** (1.27)

Note: ***, ** and * denote significant at 1%, 5% and 10%; Standard errors are in parentheses.Where HFIAS = Household Food Insecurity Access Score.WPI = Water Poverty Index.

Despite this, rural households in South Africa that implemented UCs in their farming operation achieved a higher level of water and food security than those that did not. Table 5 shows that household heads in Limpopo, Mpumalanga and Nort West provinces who adopted UCs saw a 25.18 (21%), 31.03 (26%) and 28.77 (24%) rise in WPI compared to those who did not embrace UCs, respectively. Non-adopters of UCs in Limpopo, Mpumalanga and Nort West provinces would have resulted in a subsequent rise in WPI by 22.89 (17%), 33.52 (27%) and 23.04 (19%) assuming they implement UCs in their farms, respectively. The results demonstrate that implementing UCs significantly increases the WPI of rural farming households in South Africa.

The findings align with^17,46,101^, which revealed that adopting underutilized crops especially legumes improved the water and food security of rural farmers in sub-Saharan Africa (SSA). Furthermore, the findings indicate that adopting UCs has had a significant effect on reducing the level of water poverty among rural farming households in South Africa. Furthermore, adopting UCs in farming operations in Limpopo, Mpumalanga and North West provinces of South Africa reduces the likelihood of experiencing food inadequacy (HFIAS) by 4.45 (29%), 5.05 (25%) and 4.78 (28%), respectively. If non-adopters had embraced UCs on their farm in Limpopo, Mpumalanga and North West provinces, the household food insecurity gap (HFIAS) would have decreased by 7.71 (34%), 6.27 (37%) and 5.96 (28%), respectively.

Consistently implementing UCs on the farm enhances the resilience of ecosystems, reducing the vulnerability of smallholder farmers to extreme weather events and other climate-related challenges^16,17,41^. It also maximizes agricultural productivity and minimizes the risk of food insufficiency^41^. Notably, implementing UCs enhances the ability of agriculture to withstand climate-related difficulties, while rural farm families are more effectively equipped to sustain consistent food production despite fluctuations in environment-related issues^40^. Therefore, the study’s findings validated that implementing UCs had a significant and positive impact on enhancing the water and nutritional security of households, as seen by improvements in the water poverty index and household food insecurity access score of the adopting rural households.

## Conclusion and policy recommendations

The need for a comprehensive solution to global food and nutrition security in the face of climate change is urgent. A promising yet underexplored solution lies in Africa’s underutilized crops (UCs), which have been historically overlooked despite their significant potential in addressing climate change, water scarcity, and food insecurity. These indigenous crops, inherently resilient to harsh climatic conditions, are well-suited to thrive in marginal soils with minimal water and nutrient inputs, making them a sustainable alternative to resource-intensive agricultural systems. Their cultivation could enhance food sovereignty and reduce environmental degradation while ensuring more stable food production in climate-vulnerable regions. This study documented 21 UCs – grain (5), vegetable species (6), and fruits (9), classified among 14 families and parts used for different purposes. To examine the impacts of UCs on food and water insecurity, MESR technique was employed, focusing on rural households in Limpopo, Mpumalanga, and North-West provinces. The results indicate that households adopting UCs experienced an increase in WPI by 21% (25.18), 26% (31.03), and 24% (28.77) in Limpopo, Mpumalanga, and North West provinces, respectively, compared to non-adopters. Moreover, a counterfactual analysis suggests that if non-adopters had embraced UCs, their WPI scores would have risen by 17% (22.89), 27% (33.52), and 19% (23.04) in the respective provinces. Similarly, the adoption of UCs reduced household food insecurity as measured by HFIAS. Farmers who integrated UCs into their agricultural operations experienced a reduction in food inadequacy by 29% (4.45), 25% (5.05), and 28% (4.78) in Limpopo, Mpumalanga, and North West provinces, respectively. This underscores the nutritional benefits of UCs, which provide a diverse range of essential nutrients, enhance diet quality, and increase food availability year-round. The study further revealed that age, farm size, and institutional factors, including access to extension services, climate information, and credit, as well as perceived benefits and available information sources, significantly influenced UC adoption among rural farmers. These heterogeneous factors distinguish adopters, who tend to be more food- and water-secure, from non-adopters, highlighting the importance of institutional support and information dissemination in scaling up UCs adoption. The study revealed that rural farmers in South Africa who integrated UCs into their agricultural systems achieved better food and water security outcomes than those relying solely on conventional crops. To fully harness the potential of these crops, it is crucial to invest in research and development, create supportive policies, and increase awareness among farmers. Enhancing infrastructure, fostering collaboration, and integrating climate-smart agricultural practices will further promote the adoption and success of the UCs. This could lead to more resilient agricultural systems, greater food sovereignty, and improved livelihoods for local communities.

## Electronic supplementary material

Below is the link to the electronic supplementary material.

Supplementary Information.

## Data Availability

The datasets used and/or analysed during the current study are available from the corresponding author on reasonable request.

## Appendix:

See in Table (6,7and8)

**Table 6:** Test of validity of the selection instrument

Variables	**Limpopo**		**Mpumalanga**		**North West**	
	**Coefficient**	**Robust SE**	**Coefficient**	**Robust SE**	**Coefficient**	**Robust SE**
**Socioeconomic variables**						
Age	-0.0247	0.0057	0.0136	0.0265	-0.0663	0.0768
Household size	0.1541	0.0353	-0.0139***	0.0016	0.3225*	0.1470
Marital status of the household head	0.2610	0.1514	0.1207***	0.0112	0.0674	0.0512
Educational status of HH	0.2019	0.1073	-0.0238	0.4341	0.0509**	0.0261
Years of experience	0.1275	0.1091	0.1145	0.1062	0.0761	0.0584
Land ownership	0.3124	0.5602	0.0584***	0.0100	0.0788	0.1164
Source of financing farm operation	0.4316	0.1702	0.0285	0.1062	0.0047	0.0081
**Interested variables**						
Off-farm income	0.0331	0.0245	0.0682	0.1772	-0.1159**	0.0469
Total farm size for other crops cultivation	0.0729*	0.3092	0.1009*	0.0534	0.3773***	0.0644
Total land used for UCs cultivation	0.8743	0.7530	0.1968	0.0164	0.0181	0.0162
Enterprise diversification	0.1387	0.2691	0.0892***	0.0042	0.0783***	0.0058
Membership of cooperative society	0.5421	0.5022	0.1175	0.4405	0.6217**	0.2361
**Institutional variables**						
Information on climate change	-0.1497	0.6033	-0.0102	0.0283	-0.0231	0.0580
Contact with extension agents	0.3285	1.0483	0.0391	0.0570	0.5168***	0.1141
Perceived benefit of UCs	0.0867***	0.0311	0.0707***	0.0292	0.1277***	0.0614
**Information source**						
Local newspaper/community meeting	0.0186	0.1117	0.0579	0.1257	0.0393	0.0358
Flyers/Posters	0.2507	0.2621	-0.0203	0.2569	0.0767	0.0534
Radio	0.1932*	0.0984	0.0655	0.1415	0.0797	0.0549
Smartphone	-0.2486	0.0105	0.0799	0.2307	0.0554*	0.0305
Constant	0.1505***	0.0563	0.1007***	0.0369	-0.1703***	0.0491

Note: ***, ** and * denotes significant at 1%, 5% and 10% respectively.Non-adopter of UCs is the reference category.

**Table 7:** Second stage of MESR: WPI outcome.

Variables	**Limpopo**		**Mpumalanga**		**North West**	
	**Coefficient**	**Robust SE**	**Coefficient**	**Robust SE**	**Coefficient**	**Robust SE**
**Socioeconomic variables**						
Age	0.0271***	0.0111	0.1005***	0.0047	0.1115	0.1143
Household size	0.1231***	0.0301	0.1861	0.1044	0.0895***	0.0241
Marital status of the household head	0.0072	0.0181	-0.9151	0.5031	0.1153**	0.0703
Educational status of HH	-0.1984***	0.0320	0.0164**	0.0089	0.0191**	0.0052
Years of experience	0.0128	0.0357	0.0393	0.0002	-0.0231	0.0580
Land ownership	0.0781	0.0523	0.0767	0.0577	0.5168***	0.1141
Source of financing farm operation	0.0508	0.0547	0.0797	0.0311	0.1277***	0.0614
**Interested variables**						
Off-farm income	-0.1946***	0.0489	-0.1703***	0.0022	-0.1287	0.1169
Total farm size for other crops cultivation	0.0283	0.0793	0.0145	0.0025	0.1091	0.1644
Total land used for UCs cultivation	0.0165	0.0168	-0.0147	0.0442	0.2321	0.1079
Enterprise diversification	-0.0083	0.0366	-0.0095	0.1199	0.1942	0.1740
Membership of cooperative society	0.0128	0.0357	0.0393	0.0104	0.0089	0.3495
**Institutional variables**						
Information on climate change	0.3285	1.7328	0.1222	0.3590	0.0756***	0.0294
Contact with extension agents	0.0145	0.6035	0.6304**	0.3848	0.1402	0.3865
Perceived benefit of UCs	0.0580	0.0744	-0.1920	0.1704	-0.0687	0.1711
**Information source**						
Local newspaper/community meeting	-0.0231	0.0580	-0.0001	0.0028	-0.0039	0.0028
Flyers/Posters	0.5168***	0.1141	0.0093	0.0060	0.0099	0.0060
Radio	0.1277***	0.0614	0.0128	0.0357	0.0393	0.0358
Smartphone	0.0593	0.1153	0.0781	0.0523	0.0767	0.0534
Sigma_0; Sigma_1	0.2947**	0.1075	0.1222*	0.0590	0.2967***	0.1053
Rho_0; Rho_1	0.1842**	0.0976	0.6304**	0.3848	0.2696***	0.0348
Constant	-0.1287	0.1169	0.0508***	0.0047	0.0797**	0.0349

Note: ***, ** and * denotes significant at 1%, 5% and 10% respectively.

**Table 8:** Second stage of MESR: HFIAS outcome.

Variables	**Limpopo**		**Mpumalanga**		**North West**	
	**Coefficient**	**Robust SE**	**Coefficient**	**Robust SE**	**Coefficient**	**Robust SE**
**Socioeconomic variables**						
Age	-0.0667	0.2879	0.2751	0.2095	-0.0621	0.0909
Household size	0.1389**	0.0791	0.1014	0.2496	0.1172**	0.0212
Marital status of the household head	0.2490	0.2027	0.2020	0.4210	0.0406**	0.0255
Educational status of HH	0.2085**	0.0938	0.5431**	0.4678	0.2310	0.1179
Years of experience	0.0822	0.2400	0.3126	0.5727	0.1270	0.1554
Land ownership	-0.0558	0.1167	-0.2492	0.2660	-0.7761*	0.3812
Source of financing farm operation	0.5122**	0.2550	0.0841	0.1275	0.2138	0.2372
**Interested variables**						
Off-farm income	-0.1378	0.3720	0.3223	0.2041	0.5837***	0.5044
Total farm size for other crops cultivation	0.1219	0.3824	0.1288	0.2688	-0.4824***	0.0815
Total land used for UCs cultivation	0.1038	0.5540	0.4933**	0.1190	0.0295	0.0440
Enterprise diversification	0.1280	0.3414	0.2736	0.9310	0.0128	0.0357
Membership of cooperative society	-0.1735	0.1172	0.0781	0.0523	0.0781	0.0523
**Institutional variables**						
Information on climate change	0.0767***	0.0304	0.0508	0.0547	0.0767	0.0520
Perceived benefit of UCs	-0.1946***	0.0489	0.1491	1.7406	0.0756***	0.0294
**Information source**						
Local newspaper/community meeting	0.5173***	0.0656	0.1964	0.6521	0.3192***	0.1006
Flyers/Posters	0.0284	0.0220	0.3461	0.7190	0.4123***	0.0824
Radio	-0.0487	0.0884	-0.2786*	0.1067	-0.0083	0.1117
Smartphone	0.1914	0.5003	0.4701	0.4217	0.2071	0.1268
Sigma_0; Sigma_1	0.4682***	0.0971	0.1831*	0.0971	-0.2762	0.1972
Rho_0; Rho_1	0.0491*	0.0262	-0.0562***	0.0214	0.0863**	0.0433
Constant	0.0700***	0.0048	0.6419*	0.2494	-0.2741*	0.1105

Note: ***, ** and * denotes significant at 1%, 5% and 10% respectively.
