# Supplementary material for: Leveraging Africa’s underutilized crops to combat climate change, water scarcity, and food insecurity in South Africa
Source: Sci Rep. 2025 Jun 3;15:19404. doi: 10.1038/s41598-025-03853-4 (PMC12134104; doi:10.1038/s41598-025-03853-4)
Supplement: Supplementary file 1 — Supplementary Information. [file 41598_2025_3853_MOESM1_ESM.docx]

**Table A1.** Test of validity of the selection instrument

| Variables | Limpopo | | Mpumalanga | | North West | |
| --- | --- | --- | --- | --- | --- | --- |
|  | **Coefficient** | **Robust SE** | **Coefficient** | **Robust SE** | **Coefficient** | **Robust SE** |
| Socioeconomic variables |  |  |  |  |  |  |
| Age | -0.0247 | 0.0057 | 0.0136 | 0.0265 | -0.0663 | 0.0768 |
| Household size | 0.1541 | 0.0353 | -0.0139*** | 0.0016 | 0.3225* | 0.1470 |
| Marital status of the household head | 0.2610 | 0.1514 | 0.1207*** | 0.0112 | 0.0674 | 0.0512 |
| Educational status of HH | 0.2019 | 0.1073 | -0.0238 | 0.4341 | 0.0509** | 0.0261 |
| Years of experience | 0.1275 | 0.1091 | 0.1145 | 0.1062 | 0.0761 | 0.0584 |
| Land ownership | 0.3124 | 0.5602 | 0.0584*** | 0.0100 | 0.0788 | 0.1164 |
| Source of financing farm operation | 0.4316 | 0.1702 | 0.0285 | 0.1062 | 0.0047 | 0.0081 |
| Interested variables |  |  |  |  |  |  |
| Off-farm income | 0.0331 | 0.0245 | 0.0682 | 0.1772 | -0.1159** | 0.0469 |
| Total farm size for other crops cultivation | 0.0729* | 0.3092 | 0.1009* | 0.0534 | 0.3773*** | 0.0644 |
| Total land used for UCs cultivation | 0.8743 | 0.7530 | 0.1968 | 0.0164 | 0.0181 | 0.0162 |
| Enterprise diversification | 0.1387 | 0.2691 | 0.0892*** | 0.0042 | 0.0783*** | 0.0058 |
| Membership of cooperative society | 0.5421 | 0.5022 | 0.1175 | 0.4405 | 0.6217** | 0.2361 |
| Institutional variables |  |  |  |  |  |  |
| Information on climate change | -0.1497 | 0.6033 | -0.0102 | 0.0283 | -0.0231 | 0.0580 |
| Contact with extension agents | 0.3285 | 1.0483 | 0.0391 | 0.0570 | 0.5168*** | 0.1141 |
| Perceived benefit of UCs | 0.0867*** | 0.0311 | 0.0707*** | 0.0292 | 0.1277*** | 0.0614 |
| Information source |  |  |  |  |  |  |
| Local newspaper/community meeting | 0.0186 | 0.1117 | 0.0579 | 0.1257 | 0.0393 | 0.0358 |
| Flyers/Posters | 0.2507 | 0.2621 | -0.0203 | 0.2569 | 0.0767 | 0.0534 |
| Radio | 0.1932* | 0.0984 | 0.0655 | 0.1415 | 0.0797 | 0.0549 |
| Smartphone | -0.2486 | 0.0105 | 0.0799 | 0.2307 | 0.0554* | 0.0305 |
| Constant | 0.1505*** | 0.0563 | 0.1007*** | 0.0369 | -0.1703*** | 0.0491 |

Note: ***, ** and * denotes significant at 1%, 5% and 10% respectively

Non-adopter of UCs is the reference category

**Table A2**. Second stage of MESR: WPI outcome

| Variables | Limpopo | | Mpumalanga | | North West | |
| --- | --- | --- | --- | --- | --- | --- |
|  | **Coefficient** | **Robust SE** | **Coefficient** | **Robust SE** | **Coefficient** | **Robust SE** |
| Socioeconomic variables |  |  |  |  |  |  |
| Age | 0.0271*** | 0.0111 | 0.1005*** | 0.0047 | 0.1115 | 0.1143 |
| Household size | 0.1231*** | 0.0301 | 0.1861 | 0.1044 | 0.0895*** | 0.0241 |
| Marital status of the household head | 0.0072 | 0.0181 | -0.9151 | 0.5031 | 0.1153** | 0.0703 |
| Educational status of HH | -0.1984*** | 0.0320 | 0.0164** | 0.0089 | 0.0191** | 0.0052 |
| Years of experience | 0.0128 | 0.0357 | 0.0393 | 0.0002 | -0.0231 | 0.0580 |
| Land ownership | 0.0781 | 0.0523 | 0.0767 | 0.0577 | 0.5168*** | 0.1141 |
| Source of financing farm operation | 0.0508 | 0.0547 | 0.0797 | 0.0311 | 0.1277*** | 0.0614 |
| Interested variables |  |  |  |  |  |  |
| Off-farm income | -0.1946*** | 0.0489 | -0.1703*** | 0.0022 | -0.1287 | 0.1169 |
| Total farm size for other crops cultivation | 0.0283 | 0.0793 | 0.0145 | 0.0025 | 0.1091 | 0.1644 |
| Total land used for UCs cultivation | 0.0165 | 0.0168 | -0.0147 | 0.0442 | 0.2321 | 0.1079 |
| Enterprise diversification | -0.0083 | 0.0366 | -0.0095 | 0.1199 | 0.1942 | 0.1740 |
| Membership of cooperative society | 0.0128 | 0.0357 | 0.0393 | 0.0104 | 0.0089 | 0.3495 |
| Institutional variables |  |  |  |  |  |  |
| Information on climate change | 0.3285 | 1.7328 | 0.1222 | 0.3590 | 0.0756*** | 0.0294 |
| Contact with extension agents | 0.0145 | 0.6035 | 0.6304** | 0.3848 | 0.1402 | 0.3865 |
| Perceived benefit of UCs | 0.0580 | 0.0744 | -0.1920 | 0.1704 | -0.0687 | 0.1711 |
| Information source |  |  |  |  |  |  |
| Local newspaper/community meeting | -0.0231 | 0.0580 | -0.0001 | 0.0028 | -0.0039 | 0.0028 |
| Flyers/Posters | 0.5168*** | 0.1141 | 0.0093 | 0.0060 | 0.0099 | 0.0060 |
| Radio | 0.1277*** | 0.0614 | 0.0128 | 0.0357 | 0.0393 | 0.0358 |
| Smartphone | 0.0593 | 0.1153 | 0.0781 | 0.0523 | 0.0767 | 0.0534 |
| Sigma_0; Sigma_1 | 0.2947** | 0.1075 | 0.1222* | 0.0590 | 0.2967*** | 0.1053 |
| Rho_0; Rho_1 | 0.1842** | 0.0976 | 0.6304** | 0.3848 | 0.2696*** | 0.0348 |
| Constant | -0.1287 | 0.1169 | 0.0508*** | 0.0047 | 0.0797** | 0.0349 |

Note: ***, ** and * denotes significant at 1%, 5% and 10% respectively

**Table A3**. Second stage of MESR: HFIAS outcome

| Variables | Limpopo | | Mpumalanga | | North West | |
| --- | --- | --- | --- | --- | --- | --- |
|  | **Coefficient** | **Robust SE** | **Coefficient** | **Robust SE** | **Coefficient** | **Robust SE** |
| Socioeconomic variables |  |  |  |  |  |  |
| Age | -0.0667 | 0.2879 | 0.2751 | 0.2095 | -0.0621 | 0.0909 |
| Household size | 0.1389** | 0.0791 | 0.1014 | 0.2496 | 0.1172** | 0.0212 |
| Marital status of the household head | 0.2490 | 0.2027 | 0.2020 | 0.4210 | 0.0406** | 0.0255 |
| Educational status of HH | 0.2085** | 0.0938 | 0.5431** | 0.4678 | 0.2310 | 0.1179 |
| Years of experience | 0.0822 | 0.2400 | 0.3126 | 0.5727 | 0.1270 | 0.1554 |
| Land ownership | -0.0558 | 0.1167 | -0.2492 | 0.2660 | -0.7761* | 0.3812 |
| Source of financing farm operation | 0.5122** | 0.2550 | 0.0841 | 0.1275 | 0.2138 | 0.2372 |
| Interested variables |  |  |  |  |  |  |
| Off-farm income | -0.1378 | 0.3720 | 0.3223 | 0.2041 | 0.5837*** | 0.5044 |
| Total farm size for other crops cultivation | 0.1219 | 0.3824 | 0.1288 | 0.2688 | -0.4824*** | 0.0815 |
| Total land used for UCs cultivation | 0.1038 | 0.5540 | 0.4933** | 0.1190 | 0.0295 | 0.0440 |
| Enterprise diversification | 0.1280 | 0.3414 | 0.2736 | 0.9310 | 0.0128 | 0.0357 |
| Membership of cooperative society | -0.1735 | 0.1172 | 0.0781 | 0.0523 | 0.0781 | 0.0523 |
| Institutional variables |  |  |  |  |  |  |
| Information on climate change | 0.0767*** | 0.0304 | 0.0508 | 0.0547 | 0.0767 | 0.0520 |
| Perceived benefit of UCs | -0.1946*** | 0.0489 | 0.1491 | 1.7406 | 0.0756*** | 0.0294 |
| Information source |  |  |  |  |  |  |
| Local newspaper/community meeting | 0.5173*** | 0.0656 | 0.1964 | 0.6521 | 0.3192*** | 0.1006 |
| Flyers/Posters | 0.0284 | 0.0220 | 0.3461 | 0.7190 | 0.4123*** | 0.0824 |
| Radio | -0.0487 | 0.0884 | -0.2786* | 0.1067 | -0.0083 | 0.1117 |
| Smartphone | 0.1914 | 0.5003 | 0.4701 | 0.4217 | 0.2071 | 0.1268 |
| Sigma_0; Sigma_1 | 0.4682*** | 0.0971 | 0.1831* | 0.0971 | -0.2762 | 0.1972 |
| Rho_0; Rho_1 | 0.0491* | 0.0262 | -0.0562*** | 0.0214 | 0.0863** | 0.0433 |
| Constant | 0.0700*** | 0.0048 | 0.6419* | 0.2494 | -0.2741* | 0.1105 |

Note: ***, ** and * denotes significant at 1%, 5% and 10% respectively
